# Urinary estrogens as a non-invasive biomarker of viable pregnancy in the giant panda (*Ailuropoda melanoleuca*)

**DOI:** 10.1038/s41598-019-49288-6

**Published:** 2019-09-04

**Authors:** Kirsten S. Wilson, Jella Wauters, Iain Valentine, Alan McNeilly, Simon Girling, Rengui Li, Desheng Li, Hemin Zhang, Mick T. Rae, Forbes Howie, Ruth Andrew, William Colin Duncan

**Affiliations:** 1 MRC Centre for Reproductive Health, Queen’s Medical Research Institute, University of Edinburgh, 47 Little France Crescent, Edinburgh, EH16 4TJ UK; 2 Laboratory of Chemical Analysis, Department of Veterinary Public Health and Food Safety, Faculty of Veterinary Medicine, Ghent University, 9820 Merelbeke, Belgium; 3 Pairi Daiza Foundation, Domaine de Cambron, 7940 Brugelette, Belgium; 4 Leibniz Institute for Zoo and Wildlife Research, Department Reproduction Biology, PF 700430, 10324 Berlin, Germany; 5 RZSS Edinburgh Zoo, 134 Corstorphine Road, Edinburgh, EH12 6TS UK; 6 China Conservation and Research Centre for the Giant Panda (CCRCGP), DuJiangYan City, Sichuan Province China; 7 School of Applied Sciences, Edinburgh Napier University, Sighthill Campus, Edinburgh, EH11 4BN UK; 8 BHF/University of Edinburgh Centre for Cardiovascular Science, Queen’s Medical Research Institute, 47 Little France Crescent, Edinburgh, EH16 4TJ UK

**Keywords:** Hormones, Reproductive biology, Animal physiology

## Abstract

Female giant pandas show complex reproductive traits, being seasonally monoestrus, displaying a variable length embryonic diapause and exhibiting pseudopregnancy. Currently, there is no confirmatory non-invasive biomarker of blastocyst implantation or pregnancy. This study aimed to monitor urinary estrogens across gestation in pregnancy (n = 4), pseudopregnancy (n = 4) and non-birth cycles (n = 5) in the giant panda. A pregnancy-specific profile of estrogens corrected for urinary specific gravity was identified during the gestation period. Pregnant females showed increasing concentrations of estrogens for 29 days until birth, no increase was observed during pseudopregnancy and the two profiles were distinguishable from each other for the final 2 weeks of the cycle suggesting the estrogens are of placental origin. This allowed a nomogram, starting at a known fixed point during the cycle, to be created and tested with cycles of known outcome, and cycles which were inseminated but did not result in a birth. Non-birth profiles showed deviations from that of pregnancy. We believe these deviations indicate the point of failure of the placenta to support a developing cub. Non-invasive longitudinal monitoring of estrogen concentrations therefore has the potential to be developed as a panda pregnancy test to predict viable cub development.

## Introduction

Although conservation success recently downgraded giant pandas from endangered to vulnerable^1^, there are fewer than 2000 giant pandas (*Ailuropoda melanoleuca*) in the wild throughout China. The captive breeding programme has facilitated conservation and succeeded in increasing the global population of giant pandas^2^, which includes over 500 pandas in captivity. It has also been vital in aiding understanding of their complex reproductive physiology.

Female giant pandas are seasonally monoestrus, ovulating once a year, typically between February and May^3^, following a 7–14 day follicular phase. The follicular phase is identified by increasing urinary estrogens and estrus-associated behaviours, which include scent marking, decreased appetite, lordosis and increased vocalisations^4^. Ovulation is determined when estrogens decrease from peak concentrations. After ovulation there is a biphasic luteal phase, characterised by urinary progesterone (P4) concentrations, consisting of a ‘primary P4 rise’ of 61–122 days, and a ‘secondary P4 rise’ of around 45 days^5^. During the primary P4 rise in conceiving females, embryonic diapause occurs whereby the blastocyst remains in an arrested developmental state^6^. Neither the driver for the timing nor the signal for blastocyst reactivation or implantation is known, however implantation is believed to occur during the secondary P4 rise. The peak P4 in the secondary rise is associated with a prostaglandin surge and there is a marked spike of urinary 13,14,dihydro-15-keto- prostaglandin F_2α_ (PGFM) in the urine^7^. After this, urinary P4 decreases and birth is expected within three weeks^7^.

However, giant pandas also undergo pseudopregnancy; the luteal phase of all ovulating female pandas displays the same biphasic P4 profile and urinary PGFM surge during the secondary rise, independent to pregnancy^6^. Therefore, detecting a true ongoing pregnancy in giant pandas can be challenging.

Fetal detection through ultrasound has proven successful in some cases^4,6,8^, however the procedure requires cooperation from the panda which is often challenging during the final weeks of the cycle. Urinary ceruloplasmin has been suggested as a marker of successful conception^9^ but it is primarily present during the primary P4 rise. During the secondary P4 rise, PGFM can be used as a predictive marker for the timing of birth^7^.

Estrogens have been described as remaining low and at baseline concentrations in the giant panda luteal phase^4,10,11^. However in other species estrogens play a role in blastocyst implantation^12^, maternal recognition of pregnancy^13,14^, are produced by the placenta^14–18^, and play a role in preparation for parturition^19^.

We hypothesised that estrogens are not products of the corpus luteum (CL) in giant pandas and concentrations would be different in pregnancy when compared to pseudopregnancy. Our aim was to assess urinary estrogens across the luteal phase in giant pandas with an interest in the potential period of gestation, studying pregnancy, pseudopregnancy and non-birthing inseminated estrous cycles.

## Results

### Estrous cycle hormones of the giant panda

**Figure 1:**
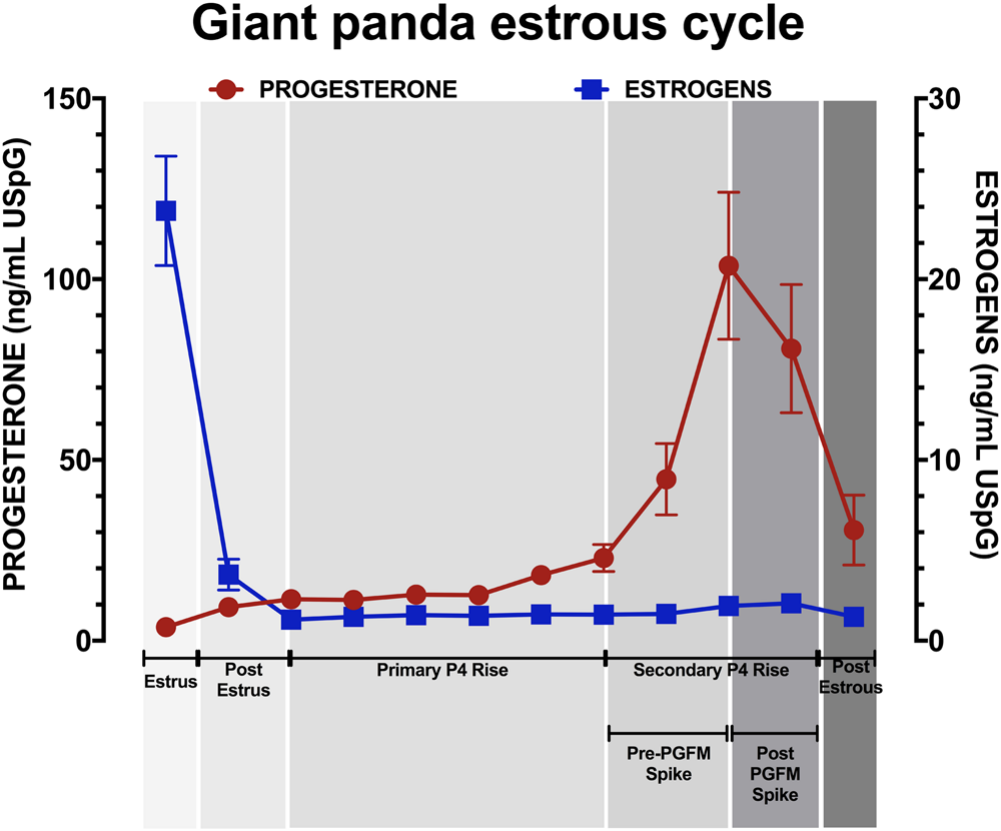
The average estrogen and progesterone concentrations ± Standard Error of the Mean (SEM) for all cycles (n = 13) from 2 weeks pre-estrus to 2 weeks post-estrous corrected for Urinary Specific Gravity (USpG). Cycles are presented as 10% periods of the whole estrous length to account for the varying cycle lengths (range 83 to 168). The primary rise lasted for 70% of the cycle. The secondary rise lasted for 30% of the cycle, and this period was then divided into Pre- and Post-PGFM Spike periods for further analysis.

We first assessed whether luteal phase estrogen concentrations were related to CL function in the giant panda estrous cycle. The average concentrations of P4 and estrogen, corrected for urinary specific gravity (USpG) across the estrous cycle of all giant pandas in this study (n = 13) are displayed in Fig. 1. There is a clear pattern of P4 concentration across the luteal phase with a five-fold increase from the primary to the secondary rise. Unlike P4, after estrus there is not a clear pattern of estrogen concentrations (Fig. 1). P4 and estrogen concentrations did not correlate across the luteal phase of the cycle (r = −0.25, P = 0.43).

### Luteal phase comparison of pregnancy and pseudopregnancy

We next assessed whether the concentrations of luteal phase hormones could distinguish between pregnant and pseudopregnant cycles. Table 1 describes the average area under the curve (AUC) for P4 and estrogen concentration profiles across the luteal phase of pregnancy (n = 3) and pseudopregnancy (n = 3). There were no significant differences between pregnancy and pseudopregnancy for P4 for any period of the luteal phase. For estrogens however, a significant increase was observed during pregnancy between secondary P4 rise pre- and post- PGFM spike periods (P < 0.005), and also between pregnancy and pseudopregnancy secondary P4 rise post-PGFM spike periods (P < 0.01; Table 1).

**Table 1:** The mean Area Under the Curve (AUC) statistic for P4 and estrogens for the luteal phase, comparing pregnancy (n = 3) and pseudopregnancy (n = 3).

		PROGESTERONE	ESTROGENS
*Primary P4 Rise*	**PREGNANT**	14.5	13.0
	**PSEUDOPREGNANT**	16.4	18.5
*Secondary P4 Rise Pre-PGFM Spike*	**PREGNANT**	43.2	4.0^a^
	**PSEUDOPREGNANT**	21.5	1.6
*Secondary P4 Rise Post-PGFM Spike*	**PREGNANT**	105.2	32.0^a,b^
	**PSEUDOPREGNANT**	95.4	6.7^b^

There were no significant differences between pregnant and pseudopregnant P4. Estrogens were significantly increased in pregnancy between the secondary P4 rise pre- and post-PGFM spike periods, and between pregnancy and pseudopregnancy secondary P4 rise post-PGFM spike periods. The superscript letters indicate significant difference between estrous phases.

### Estrogens during the secondary progesterone rise

**Figure 2:**
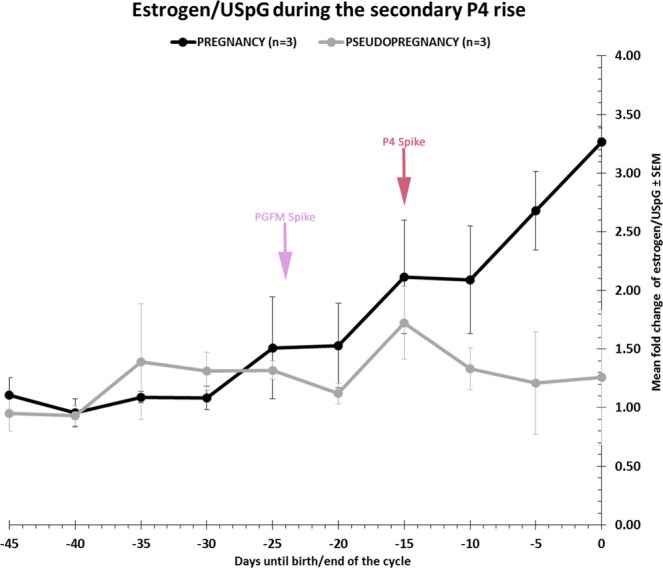
The mean fold change of estrogen/Urinary Specific Gravity (USpG) ± Standard Error of the Mean (SEM) during the secondary P4 rise in pregnancy (n = 3; solid black line) and pseudopregnancy (n = 3; solid grey line). Each point is a 5-day average, and plotted as days from birth/the end of the cycle. Birth/the end of the cycle is 0 on the x-axis. AUC analysis showed that these patterns were significantly different (P < 0.0001) following the PGFM spike.

**Figure 3:**
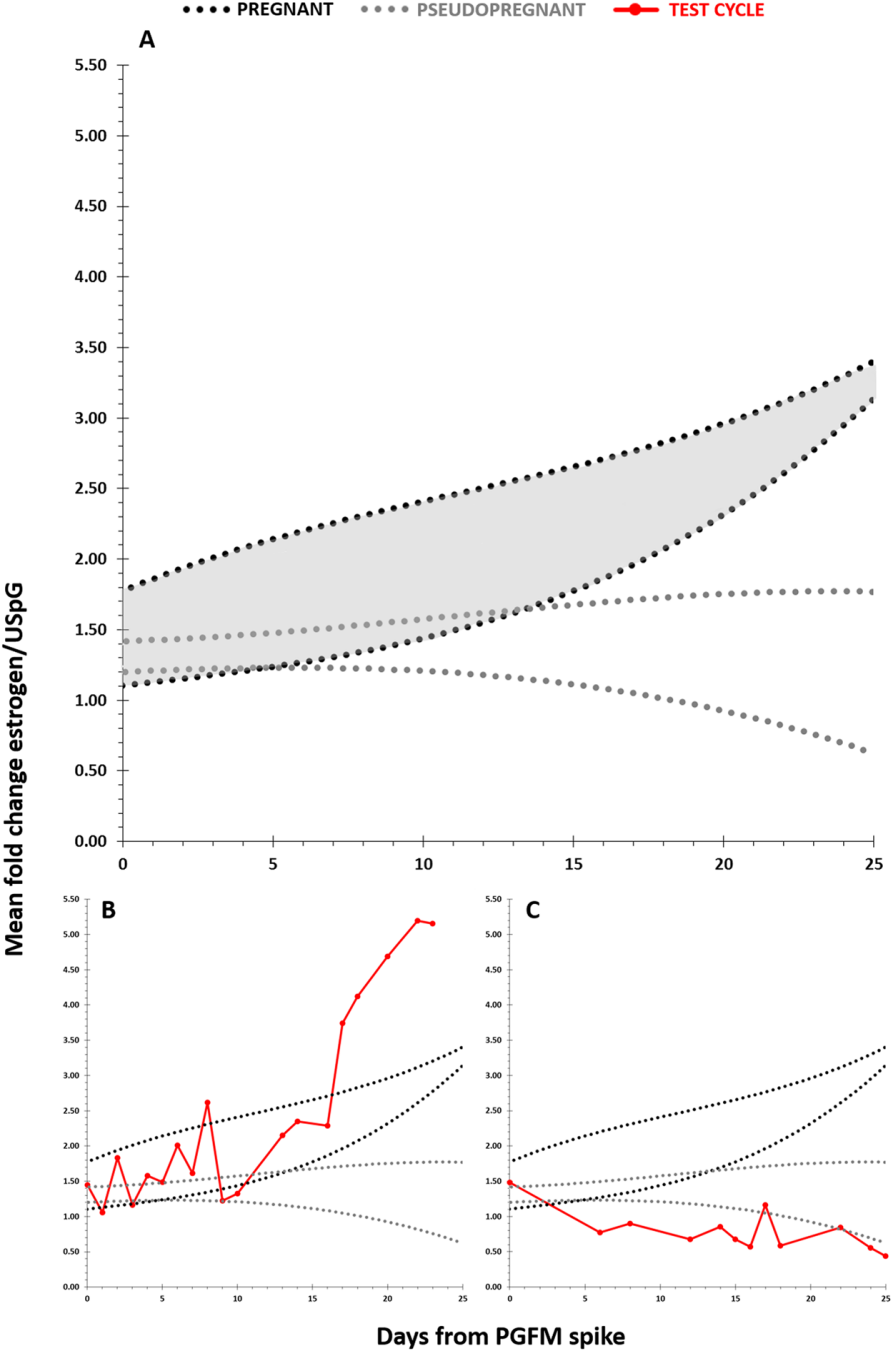
Testing two estrous cycles’ estrogen/Urinary Specific Gravity (USpG) profiles using the nomogram. A = the black dotted range indicates pregnancy, the grey dotted range indicates pseudopregnancy. B = the results from a known birth (pregnancy) cycle, C = the results from a known pseudopregnancy. The x-axis represents days from the start of the PGFM spike, with 0 defined as the last day of baseline PGFM before the spike.

As the estrogen concentrations were different in pregnant compared with pseudopregnant cycles, we examined the estrogen concentrations between secondary P4 rise pre- and post- PGFM spike periods in more detail. During the secondary P4 rise, estrogens in pregnancy showed an increasing profile for the final 29 ± 3 days of pregnancy (Fig. 2) and following the PGFM spike, estrogen concentrations increased 6 fold (Table 1). This allowed a nomogram to be developed to model the hormonal changes expected during pregnancy (Fig. 3A). This describes the Standard Error of the Mean (SEM) range of estrogen/USpG for pregnancy (black dotted range) and pseudopregnancy (grey dotted range) and is plotted from the luteal PGFM spike, a time point which is known before the outcome of pregnancy is known.

### Validation of pregnancy nomogram

Estrogen/USpG was further assessed in two further cycles with known outcomes, a pregnancy and pseudopregnancy, to validate the nomogram (Fig. 3A). In the cycle leading to a birth (Fig. 3B) the pattern was clearly in the pregnancy range and in the pseudopregnancy the pattern was that of a pseudopregnant individual (Fig. 3C).

### The nature of the estrogen rise at the end of gestation

As the rise in estrogens before birth would be consistent with a placental source of the estrogen, we examined the concentrations of estriol as it is a major placental estrogen in several species, including women^18,20^. The estriol profile was not significantly different between pregnant and pseudopregnant cycles, and concentrations appeared to decrease in the final week of all cycles (data not shown).

### Using the nomogram to interrogate inseminated non-birth cycles

**Figure 4:**
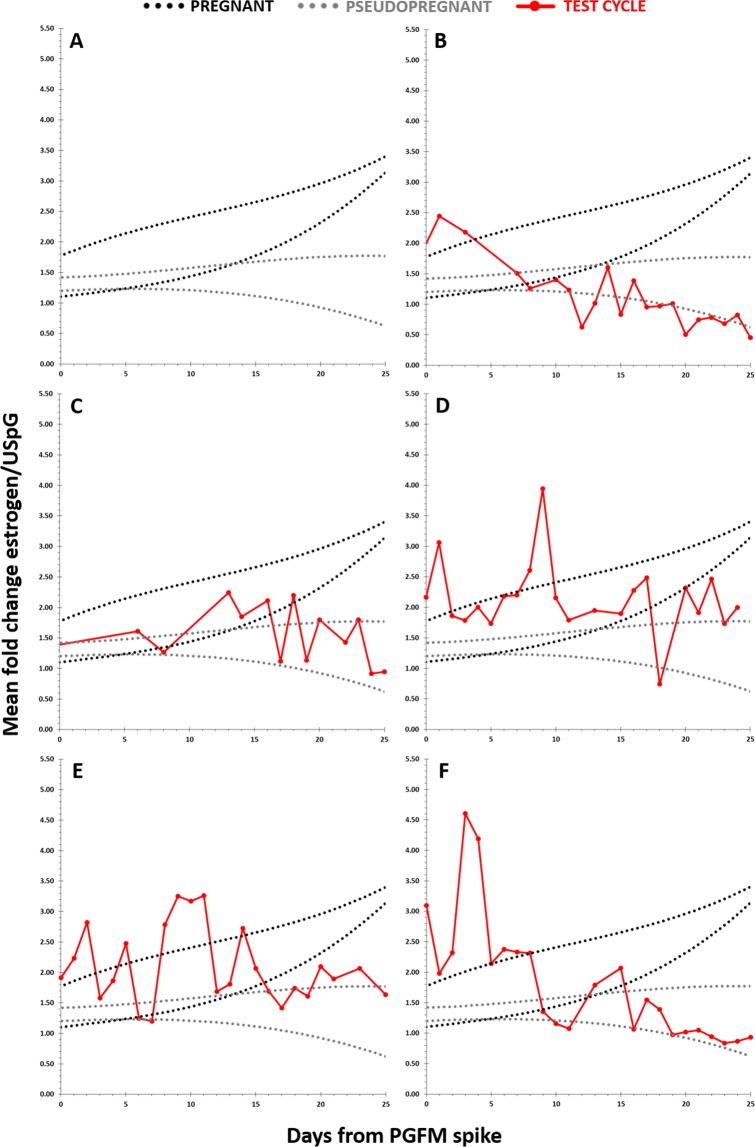
Testing five estrous cycles’, which were inseminated but non-birth cycles, estrogen/Urinary Specific Gravity (USpG) profiles using the nomogram. A = the black dotted range indicates pregnancy, the grey dotted range indicates pseudopregnancy. B-F = the red line on each nomogram represents inseminated but non-birth cycles. The x-axis represents days from the start of the PGFM spike, with 0 defined as the last day of baseline PGFM before the spike.

We examined five non-birth cycles to determine if this could highlight whether there was a pregnancy and if there was any indication at what time a pregnancy failed using the nomogram (Fig. 4A). In one cycle (Fig. 4B) there was no evidence of pregnancy. However, in four cycles (Fig. 4C–F) there were intermediate estrogen concentrations with the deviation becoming clear between 16 and 18 days after the PGFM spike.

## Discussion

Our study clearly illustrates that the measurement of urinary estrogens corrected for USpG provides noteworthy potential as a non-invasive biomarker of ongoing pregnancy in the giant panda.

Previous reports state that urinary estrogens remain unchanged for the duration of the luteal phase following estrus^4,10,11^, however we are able to identify significant changes correcting estrogens for USpG as an alternative to creatinine^21^, which has previously been used for urinary hormone correction in giant pandas. Creatinine (Cr), a muscle metabolite with fairly constant daily excretion, has been previously used as a marker of urine concentration for hormone correction in giant panda urine. However, there is a drive for pandas to increase weight during the primary P4 rise, and a decrease of activity and food intake with weight loss during the secondary P4 rise. These behavioural changes are paired with large fluctuations in Cr concentrations^21,22^. USpG can be utilised as an alternative^21^, particularly at this time, as it is affected by dietary or activity changes to a lesser extent than Cr. During the secondary P4 rise, we identified a 6-fold increase of estrogens following the initial PGFM spike in pregnancy, which lasts for 29 ± 3 days, until birth. Such an estrogen increase was not observed in pseudopregnant females. More particularly, the profiles of pregnancy and pseudopregnancy were able to be clearly resolved from each other during the final 2 weeks of the cycle.

The biphasic P4 profile cannot diagnose pregnancy as P4 originates from the corpus luteum (CL) which is present in all cyclic female pandas^23^. It has been previously shown that estrogens are low in abundance in the luteal phase^4,10,11^ and we have confirmed this. Nevertheless, in identifying no correlation between P4 and estrogen across the luteal phase, we suggest that the estrogens are not produced by the CL.

In contrast, we suggest that the increasing urinary estrogens measured during successful pregnancy is a biomarker of the placenta itself or of a placental product.

Giant panda placentation is suggested to be similar to that of other bears^8^, which is speculated to be endotheliochorial alike other carnivores, such as cats and dogs^24^. Production of both P4 and estrogen has been shown in the domestic cat placenta^25^, and placental steroidogenesis of estrogens has been previously suggested in the Japanese black bear^26^ (*Ursus thibetanus japonicas*) - the presence of the P450 aromatase enzyme detected in the syncytiotrophoblast of the placenta indicates the potential of the placenta to produce estrogens from androgens. The latter study also identified that the CL had only minimal capabilities to produce estrogens during embryonic diapause, suggesting that any changes of estrogens would only be detected following implantation of the blastocyst. With the suggestion of a short but rapid period of fetal growth, approximately 20 days, suggested for the giant panda^4,23^, our results, indicating a distinguishable difference between pregnant and pseudopregnant females for the final 2 weeks of the cycle, make placental steroidogenesis of estrogens plausible.

We believe the possibility of placental estrogen synthesis in the giant panda can be further corroborated by the urinary estrogen profiles of unsuccessful pregnancies as these profiles range from being indistinguishable from pseudopregnancies, to showing increasing concentrations until the final week of the luteal phase. We believe that profiles which show an initial increase in estrogen concentration then decrease are indicating the point at which fetal development stops as the placenta has stopped functioning correctly. By monitoring urinary estrogens we can indirectly determine placental function, and thus a surrogate of cub health, and therefore determine which potentially pregnant giant pandas will go on to give birth successfully.

To facilitate such predictive monitoring of panda pregnancy outcome likelihood, a nomogram was designed, supported by our study results. This has utility through determining pregnancy status in the giant panda, thereby facilitating timely establishment of appropriate husbandry preparations in pregnant females. To demonstrate its validity, we tested the nomogram successfully on two other cycles with known outcome. Following on from the validation of the nomogram, five additional cycles, where the panda was inseminated but did not give birth, were compared with this profile. From this, one cycle was deemed to be unsuccessful as it followed closely to the pseudopregnant profile. It is possible that there was no conception, or if conception did occur that implantation did not successfully take place or loss happened early in the secondary P4 rise. Four cycles appeared to be more successful as they showed a profile with concentrations within the pregnancy range and a generally increasing profile. However, the increase was not maintained until the end of the cycles, and the profiles decreased below the expected pregnancy range. We suggest that these profiles indeed indicate successful conception, blastocyst implantation and the beginning of fetal development. The end-term decrease in the profiles was indicative that birth would not occur. These examples clearly demonstrate a well-defined application potential for the nomogram tool in future prospective, or retrospective, reproductive monitoring, serving as a novel and much needed giant panda pregnancy test, in conjunction with urinary PGFM measurement.

In conclusion, correcting urinary estrogens for USpG as an alternative to Cr provides a profile with a pregnancy-specific increase, which can be used to determine success from 14 days after the PFGM spike. This increase is not identified in known pseudopregnancies, and monitoring estrogen/USpG in ongoing cycles can identify whether a female will go on to give birth or not. To the best of our knowledge, this is the first indication of changes to the estrogen profile during the luteal phase of the giant panda estrous cycle, and with no correlation to the P4 of CL origin, we believe that the estrogen increase during pregnancy is of placental origin. Having the potential to monitor placental and cub viability is a valuable tool to all facilities holding breeding giant pandas. Therefore, we would welcome additional validation and refinement of the nomogram to improve prediction of birth in inseminated or mated cycles.

## Materials and Methods

### Sample collection

Urine samples were collected from five female giant pandas housed at RZSS Edinburgh Zoo, Scotland (SB569), Pairi Daiza, Belgium (SB741), Smithsonian’s National Zoological Park, Washington DC, USA (SB473), Ouwehands Dierenpark, The Netherlands (SB884), and Zoo Parc De Beauval, France (SB723). An average of 80 urine samples per cycle from thirteen ovulatory estrous cycles were available, representing 4 pregnancies, 4 true pseudopregnancies (non-mated and non-artificially inseminated (AI) cycles), and 5 inseminated but non-birth cycles (Table 2), collected between 2013 and 2018.

**Table 2:** A summary of the thirteen estrous cycles from the five females included in the study, indicating the pandas which contributed towards the pregnant, pseudopregnant and non-birth groups, and the number of urine samples analysed for each female.

Giant Panda Studbook (SB) #	# of pregnant cycles	# of pseudo-pregnant cycles	# of non-birth cycles	# of urine samples
473	2	0	0	66
569	0	1	4	539
723	1	0	0	124
741	1	1	1	221
884	0	2	0	94

This study was performed on non-invasively collected urine samples which are collected daily, where possible, for reproductive monitoring, and which were aspirated from the floor of the enclosure in the absence of the animal during routine maintenance, and frozen at −20 °C. All animal-related work was conducted in line with the relevant national and international guidelines, and no specific ethical approval was required for this study.

Following the first thaw, samples were centrifuged (15 minutes at 2000g at room temperature), and were assessed for density by USpG prior to endocrine measurements. Urine from SB723 and SB741 were analysed at Ghent University. Urine from SB473, SB569 and SB884 were analysed at the University of Edinburgh. All urine samples were transported frozen from their respective zoological parks to the universities.

### Urinary Specific Gravity (USpG)

USpG was measured as previously described^21^ using a handheld digital refractometer (ATAGO, Japan), designed either for human (PAL-10S, range 1.000–1.060) or cat urine (PAL-USG (Cat), range 1.000–1.080). The use of either refractometer for the measurement of giant panda USpG has been validated previously^21^. The same refractometer was used for all samples of the same panda – SB723, 741 and 884 samples were measured with the PAL-USG; SB473 and 569 samples were measured with the PAL-10S. Briefly, deionised water (300 µL) calibrated the instrument prior to the measurement of USpG of the sample (300 µL). Hormones were corrected for USpG as described for giant pandas^21^, making use of the following formula^27^:

$$Estrogen\,corrected\,for\,USpG=Raw\,estrogen\,concentration\,\times \,[\frac{USpG\,target-1.000}{USpG\,sample-1.000}]$$

### Measurement of estrogens

Estrogens in giant panda urine can be measured by metabolite assays for both estrone-3-glucuronide (E1G) and estrone-3-sulphate (E1S). In this study urine samples from SB473, 569 and 884 were measured only for E1G; from SB723 only for E1S; and from SB741 for both E1G and E1S, this data was used to produce a conversion factor for E1S to E1G to allow all sample results to be comparable. E1G measurements were undertaken at the MRC Centre for Reproductive Health, University of Edinburgh, while E1S measurements were undertaken at the Laboratory of Chemical Analysis, Faculty of Veterinary Medicine, Ghent University. In addition, estriol, an estrogen known to be produced by the human placenta^19,20,28^, was also measured in urine samples in the final three weeks of the cycle. A total of 91 urine samples from 3 pregnant (n = 54 samples) and 3 pseudopregnant (n = 37 samples) cycles of 4 females (SB473, 569, 741, 884) were tested for estriol.

E1G was measured by E1G DetectX® Enzyme Immunoassay (EIA) Kit (K036-H5; Arbor Assays™, Ann Arbor, Michigan, USA) following the manufacturer’s protocol. Cross reactivities of 100%, 66.6%, 238%, 7.8%, 3.8% and 3.3% are reported for E1G, E1S, estrone, 17β-estradiol, estradiol-3-glucuronide and estradiol-3-sulphate respectively. The limit of detection was 8.76 pg/mL. Briefly, prepared standards, controls and urine (50 µL) were added in duplicate to the wells of a 96-well pre-coated plate (Goat Anti-Rabbit Immunoglobulin G (IgG)). Urine was diluted by a factor of 5 in Assay Buffer prior to analysis on the assay. E1G Enzyme Conjugate and E1G Antibody (25 µL each) were added to each well. Following incubation (2 hours, room temperature) each well was washed 4 times with prepared wash buffer (300 µL) and dried on paper towels. 3,3’,5,5’-Tetramethylbenzidine (TMB; 100 µL) was added to each well for 30 minutes of incubation in the dark at room temperature. The reaction was stopped by the addition of Stop Solution (50 µL). The absorbance was quantified at 450 nm on a LT-4500 Microplate Absorbance Reader (LabTech, Version 7 2010, Tecan Group Ltd., Switzerland). EIA readings were analysed using a 4 Parameter Logistic (4PLC) nonlinear regression model on MasterPlex ReaderFit Software (Version 2; Hitachi Solutions, USA). Inter- and intra-assay coefficients of variation (CV) were calculated to be 12.5% and 4.0% respectively.

E1S was measured as described by Wauters *et al*.^21^, using the Estrone DetectX® EIA Kit (K031-H1; Arbor Assays™, Ann Arbor, Michigan, USA), replacing the estrone standard with the E1S standard (C135–125 µL; Arbor Assays™, Ann Arbor, Michigan, USA). Cross reactivities of 100%, 112%, 65.5%, and 5% are reported for estrone, E1G, E1S and estradiol respectively. The limit of detection of the assay was 28.2 pg/mL. The assay was performed following the manufacturer’s protocol as described above; however samples were diluted in Assay Buffer by a factor of 10 for analysis. Inter- and intra-assay CVs were calculated as 10.5% and 6.0% respectively. In whole cycles where only E1S was measured, the data was converted to E1G through the use of a conversion factor (0.381). This conversion factor was determined by the analysis of 93 urine samples for both E1G and E1S that were compared to allow the determination of a conversion factor from E1S to E1G. There is a highly statistically significant (P < 0.0001) linear correlation (r = 0.889) between E1G and E1S measurements and the conversion factor ensured equivalent concentrations for analysis.

Estriol was measured using the Estriol DetectX® EIA Kit (K064-H5; Arbor Assays™, Ann Arbor, Michigan, USA). Cross reactivities of 100%, 57.16%, 38.5%, 6.77%, 0.03% and 0.02% were reported for estriol, estriol-3-glucuronide, estriol-3-sulphate, 16-epiestriol, 17β-estradiol and 17-epiestriol respectively. The limit of detection of the assay was 6.81 pg/mL. The assay was performed following the manufacturer’s protocol as described above; urine samples were diluted in Assay Buffer by a factor of 2 for analysis. Inter- and intra-assay CVs were calculated as 14.2% and 11.9% respectively.

### Measurement of Progesterone

P4 was measured using the Progesterone DetectX® EIA Kit (K025-H5; Arbor Assays™, Ann Arbor, Michigan, USA). Cross reactivities of 100%, 172%, 188%, 2.7%, 127%, 7% and 5.9% were reported for P4, 3β-hydroxyprogesterone, 3-hydroxyprogesterone, 11β-hydroxyprogesterone, 11-hydroxyprogesterone, 5-dihydroxyprogesterone and pregnenolone respectively. The limit of detection of the assay was 52.9 pg/mL. The assay was performed following the manufacturer’s guidelines as described above; urine samples were diluted in Assay Buffer by a factor of 10 until the secondary P4 rise when a factor of at least 100 was required. Inter- and intra-assay CVs were calculated as 13.8% and 2.0% respectively.

### Measurement of PGFM

PGFM was measured using the PGFM DetectX® EIA Kit (K022-H5; Arbor Assays™, Ann Arbor, Michigan, USA). Cross reactivities of 100% and 1.5% were reported for PGFM and Prostaglandin E Metabolite respectively. The limit of detection of the assay was 46.2 pg/mL. The assay was performed following the manufacturer’s guidelines as described above; urine samples were diluted in Assay Buffer by a factor of 10 until peak P4 was reached, when a factor of 100 was required. Inter- and intra-assay CVs were calculated as 11.1% and 18.3% respectively.

### Data analysis

The onset of estrus was defined as peak estrogen concentrations, with ovulation defined as a decrease in estrogens and an increase in P4. The onset of the primary P4 rise was defined as starting seven days post-estrus^21^. The onset of the secondary P4 rise was defined as being when the P4 concentration on two consecutive days was two standard deviations above the mean P4 concentration^5^. In pregnant pandas, the day of parturition was defined as the final day of the cycle. For pseudopregnant or non-birth cycles the date of the end of the cycle was calculated based on the PGFM profile; the end of cycle is defined as 24 days after the last baseline measurement of PGFM before the initial PGFM spike. One pregnancy and one pseudopregnancy were randomly assigned to a test paradigm, each of the other three cycles of pregnancy and pseudopregnancy were used to model differences between cycle outcomes.

In comparing P4 and estrogen between pregnancy and pseudopregnancy, data were standardised allowing for individual variation in baseline concentrations. AUC statistics were applied to each cycle for P4 and estrogen comparison of pregnant and pseudopregnant outcomes. The baseline for the AUC statistic was calculated as the mean primary rise value for each of estrogen and P4 concentrations. The average AUC for each phase of each hormone of each cycle was then used to determine the overall AUC for each phase of each hormone for both pregnancy (n = 3) and pseudopregnancy (n = 3).

AUC statistics were also applied when comparing the pregnancy and pseudopregnancy estrogen profiles during the secondary P4 rise in the pre- and post-PGFM periods.

When comparing estrogens within the secondary P4 rise, the estrogen data was standardised to ‘mean fold change of estrogen’. The average estrogen concentration for the duration of the primary P4 rise was calculated for each cycle. Each estrogen value was then divided by this average to determine the mean fold change of the sample. This allowed measurements of either E1G or E1S to be comparable for secondary P4 rise assessment without needing a conversion factor to analyse the data.

Statistical analysis was undertaken on GraphPad Prism (Version 7.02, GraphPad Software, Inc., California, USA). Parametric, non-paired, statistics were applied based on normality of the data being found by a Shapiro-Wilk test. Pearson’s correlation coefficient was applied to assess the correlation of P4 and estrogen across the estrous cycle. A one-way ANOVA with Tukey’s Multiple Comparisons was applied to compare the different reproductive phases of pregnant and pseudopregnant outcomes. Significance was defined as P < 0.05.

## Data Availability

The datasets involved in this study can be requested from the corresponding author.
